# Bilateral Metachronous Typical and Atypical Carcinoid Tumors of the Lung

**DOI:** 10.1111/1759-7714.70078

**Published:** 2025-04-28

**Authors:** Alberto Busetto, Giovanni Maria Comacchio, Vincenzo Verzeletti, Fares Shamshoum, Francesco Fortarezza, Federica Pezzuto, Andrea Dell'Amore, Fiorella Calabrese, Federico Rea

**Affiliations:** ^1^ Department of Cardiac, Thoracic, Vascular Sciences and Public Health University of Padua Padua Italy; ^2^ Thoracic Surgery Unit, Division of Surgery University ‐ Hospital of Padua Padua Italy; ^3^ Pathology Unit, Division of Integrated Diagnostics University ‐ Hospital of Padua Padua Italy

**Keywords:** atypical carcinoid, bilateral pulmonary tumors, metachronous tumors, mini‐invasive surgery, typical carcinoid

## Abstract

Bronchial carcinoids are uncommon neuroendocrine tumors. According to their pathological differentiation, they are divided into typical and atypical forms, with diverse biological behavior and aggressiveness. Bronchial carcinoids may be associated with familial neuroendocrine syndromes, such as MEN‐1. They can also present initially as diffuse hyperplastic proliferation of neuroendocrine foci throughout the pulmonary parenchyma (DIPNECH). Metachronous and bilateral forms are sporadic in the literature. We describe a case of a 68‐year‐old man with metachronous bilateral typical‐atypical carcinoid neoplasms. The patient was treated with a two‐stage mini‐invasive pulmonary surgery in a time frame of 5 years. This case may be unique because it features two rare and distinct pathological entities in the same patient, not associated with any known genetic mutation. Carcinoid tumors require multidisciplinary care and a collaborative approach due to their pleomorphic behavior, ensuring comprehensive management and maximizing therapeutic efficacy.

## Graphical abstract

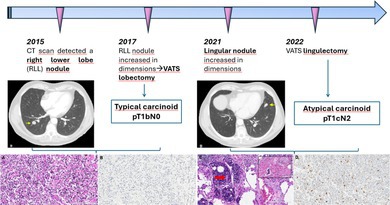

## 1 Introduction

Bronchial carcinoids are uncommon, well‐differentiated neuroendocrine tumors (NET). They can be differentiated into typical carcinoid (TC) and atypical carcinoid (AC) forms, characterized by different histology and biological behavior. Lung‐sparing surgery represents the treatment of choice [1]. Metachronous carcinoid tumors are extremely rare [2], and to our knowledge, this is the only case of metachronous bilateral typical‐atypical carcinoid yet described.

## 2 Case Report

**FIGURE 1:**
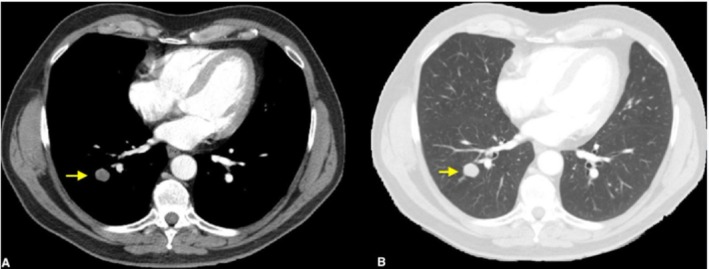
Chest CT scan showing a dense nodule of 18 × 15 mm of the right lower pulmonary lobe. (A) Mediastinal window; (B) parenchymal window.

**FIGURE 2:**
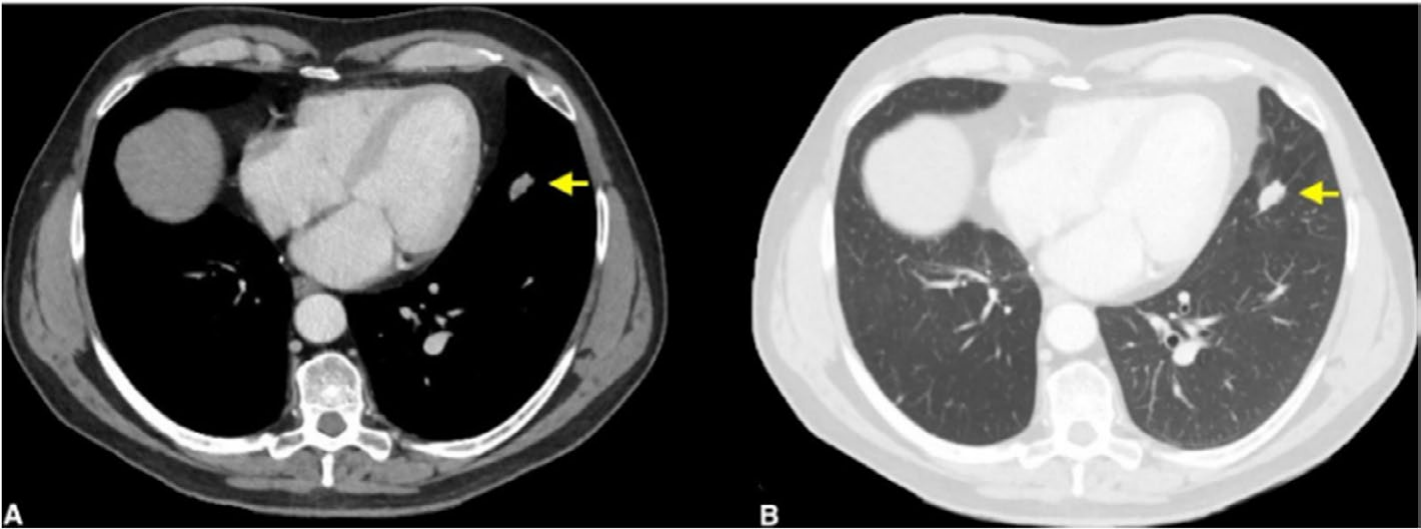
Chest CT scan showing a dense nodule of 20 × 14 mm at the inferior lingular segment. (A) Mediastinal window; (B) parenchymal window.

**FIGURE 3:**
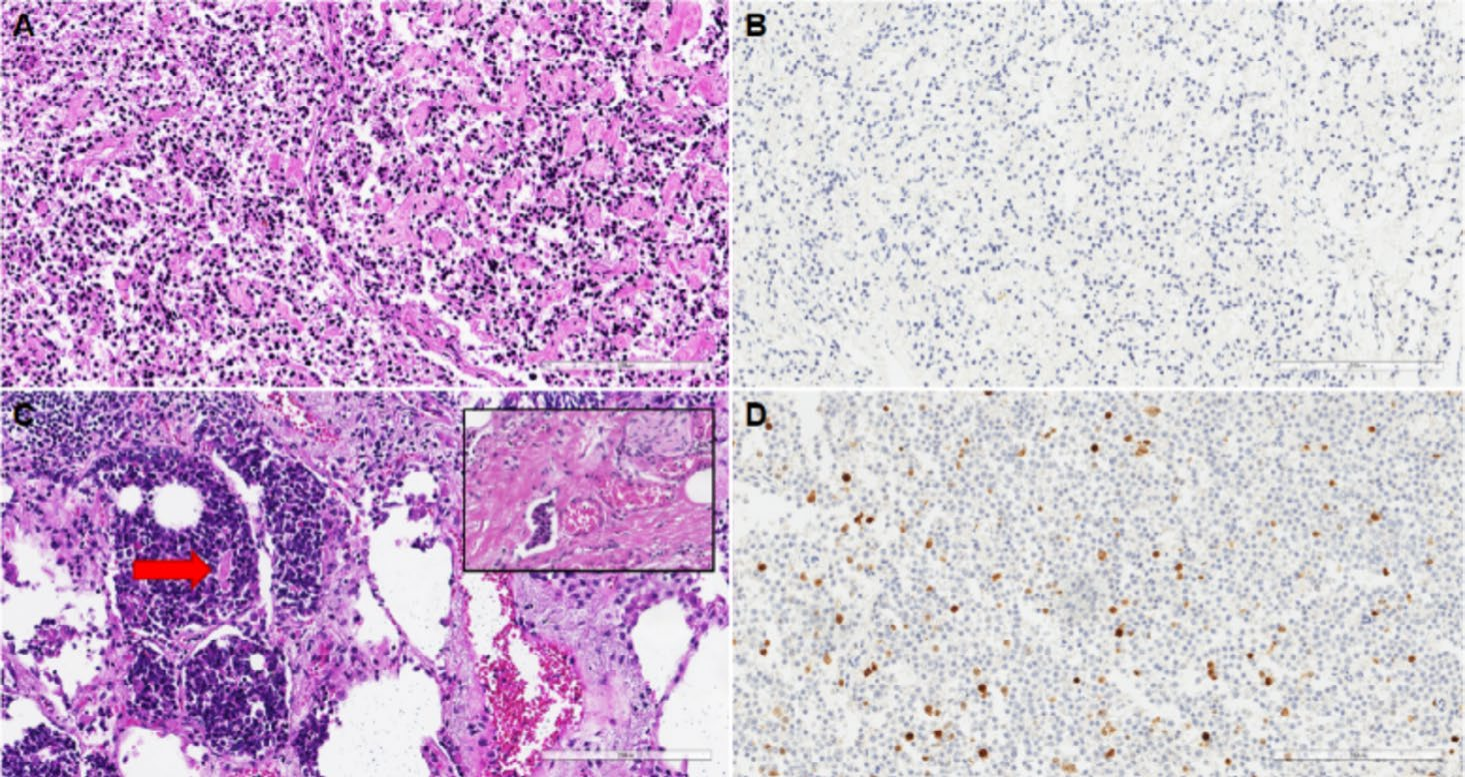
(A) Representative histological section of the typical carcinoid tumor showing small‐sized uniform cells with mild atypia (hematoxylin and eosin, 200× original magnification). (B) Proliferative index was 1% (immunostaining for Ki67, 200× original magnification). (C) In the atypical carcinoid small foci of punctate necrosis (red arrow) and vascular invasion (inset) were detected (hematoxylin and eosin, 200× original magnification). (D) Proliferative index was 15% (immunostaining for Ki67, 200× original magnification).

A 68‐year‐old male with a history of smoking underwent an abdomen computed tomography (CT) scan in 2015 as part of other investigations, during which three pulmonary nodules were detected in the right lower, middle, and lingular lobes in the cranial sequences. Subsequently, the patient underwent yearly follow‐up, during which a progressive increase in the size of a nodule in the right lower lobe was observed (Figure 1), albeit without significant uptake at Gallium‐68 DOTATOC‐Positron Emission Tomography (68Ga‐DOTATOC‐PET). In August 2017, the patient underwent d'emblée thoracoscopic right lower lobectomy and radical lymphadenectomy due to its peripheral location, which did not allow for preoperative biopsy. The final histological examination confirmed a TC staged as pT1bN0 (TNM VIII ed.). Following surgery, the patient underwent outpatient follow‐up every 6 months. In December 2021, although the middle lobe nodule remained stable over time, an increase in the size of a lingular node was detected (Figure 2), which showed 68Ga‐DOTATOC‐PET uptake. No tracer uptake was shown in intrapulmonary or mediastinal lymph nodes. The case was discussed in our multidisciplinary team; preoperative biopsy was not possible because of the central parenchymal location of the nodule, and so indication for direct surgery was given. Consequently, the patient underwent VATS lingulectomy associated with radical lymphadenectomy in July 2022 without complications. Histological examination revealed an AC staged as T1cN2 (TNM VIII ed.) with metastases in bronchial carinal and aorto‐pulmonary window lymph nodes (Figure 3). Our pathologists meticulously reviewed the anatomical specimens for other areas of neuroendocrine undifferentiation or pathological cell growth, but none were found. After a second multidisciplinary consultation, the patient was not deemed eligible for adjuvant radiotherapy or other therapies because of the single N2 station (aorto‐pulmonary window) and the time elapsed since surgery greater than 3 months. He remains free of evidence of disease to date. He continues to undergo clinical and radiological follow‐up. The patient provided written informed consent for enrollment in this study.

## 3 Discussion

Bronchial carcinoids account for 2%–5% of all pulmonary neoplasms [1]. They are well differentiated NET. TC generally has a more indolent course than AC, which exhibit a higher relapse rate. Up to 40% of carcinoids do not express somatostatin receptors and may be silent at 68‐Ga‐DOTATOC‐PET, as observed in our case [3]. Although the sensitivity of 68‐Ga‐DOTATOC‐PET is high, false negative cases may occur either in primitive carcinoid and in their lymph node metastasis, as in our AC [4]. Pulmonary carcinoids can be managed through various resection methods, such as laser therapy for central forms or surgery for peripheral lesions. Parenchyma‐sparing surgery is preferred because of the frequent recurrence of these tumors. In our case, we opted for surgical intervention for both lesions due to their peripheral localization and to ensure maximal radicality [1]. Although synchronous bilateral multiple TC have been reported in the literature, they are often misdiagnosed as metastases [5]. Metachronous TC tumors are rarer and frequently managed with nonsurgical approaches such as bronchoscopic removal or laser coagulation. Bilateral AC are exceptional but have been described in the literature [6]. This is the first case of metachronous typical‐atypical carcinoids to our knowledge. The second tumor arose contralaterally after 5 years, and after a negative follow‐up. The two tumors exhibit clearly different histological and microscopic characteristics (Table 1), and their different aggressiveness was evident in the final pathological report. Although NET typically occur as sporadic isolated entities, they may rarely manifest as part of familial syndromes, such as MEN‐1 (multiple endocrine neoplasia syndrome‐type 1). The multicentric origin of peripheral carcinoids (DIPNECH‐diffuse idiopathic pulmonary neuroendocrine cell hyperplasia) has also been documented [2]. Our patient has no family history of such conditions and all radiological examinations conducted over the years revealed no suspicious lesions elsewhere in the body. Genetic testing for mutations related to familial syndromes returned negative. The most probable hypothesis is that our patient coincidentally developed a combination of rare neoplasms or perhaps an unknown sporadic mutation predisposed him to carcinoid tumors. In conclusion, bilateral metachronous typical‐atypical carcinoids are extremely rare entities that necessitate radical surgical treatment for optimal prognosis and survival. These particular cases require multidisciplinary care by various specialists, such as surgeons, radiologists, oncologists, and radiotherapists. This collaborative approach is pivotal in addressing their pleomorphic behavior, ensuring comprehensive management tailored to individual patient needs and maximizing therapeutic efficacy.

**TABLE 1:** Comparison of the histological feature of the two metachronous tumors.

	First neoplasm (2017)	Second neoplasm (2022)
Histotype	Typical carcinoid	Atypical carcinoid
Number of mitoses (/2 mm^2^)	0	2
Neoplastic necrosis	Absent	Punctate necrosis
Lymphomonocitary infiltration/perineoplastic	≤ 10%	11%–30%
Neoplastic vascular invasion	None	Present, diffuse
Synaptophysin	−	+
Chromogranin	+	+
Ki‐67	1%	15%

## Author Contributions

Conceptualization: A.B. and G.M.C. Methodology: G.M.C., F.P., and V.V. Software: A.B. and V.V. Validation: F.S., G.M.C., and F.R. Formal analysis: F.F., F.C., and A.D.A. Investigation: A.B. Data curation: V.V., F.P., F.S., and F.R. Writing – original draft preparation: A.B., F.P., and G.M.C. Writing – review and editing: F.F., F.C., and F.R. Visualization: G.M.C. Supervision: A.D.A. and F.R. Project administration: A.D.A. All authors have read and agreed to the published version of the manuscript.

## Consent

The patient involved in this case report provided written informed consent for enrollment in this study.

## Conflicts of Interest

The authors declare no conflicts of interest.

## Data Availability

The data that support the findings of this study are available on request from the corresponding author. The data are not publicly available due to privacy.
